# Supplementary material for: Hypothalamic melanin-concentrating hormone neurons integrate food-motivated appetitive and consummatory processes in rats
Source: Nat Commun. 2023 Mar 29;14:1755. doi: 10.1038/s41467-023-37344-9 (PMC10060386; doi:10.1038/s41467-023-37344-9)
Supplement: Supplementary file 1 — Supplementary Information [file 41467_2023_37344_MOESM1_ESM.pdf]

Hypothalamic melanin-concentrating hormone neurons integrate food-motivated appetitive and consummatory processes in male rats

Subramanian et al.

Supplementary Figure 1

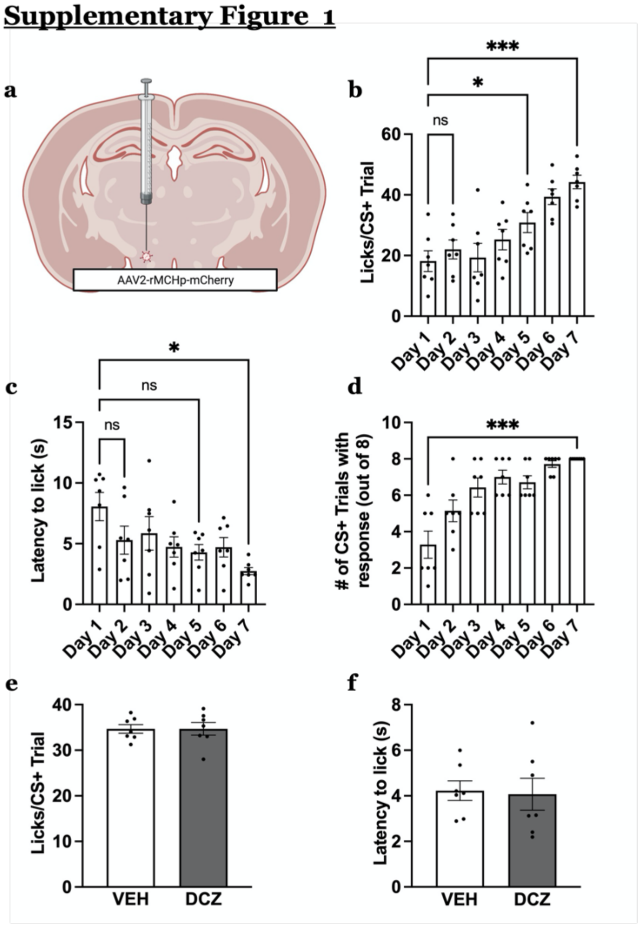

Supplementary Figure 1. IP DCZ does not impact appetitive responsivity to discrete sucrose-predictive Pavlovian cues in rats (n=7). Animals were injected using a counterbalanced, within subjects design. **a** Schematic cartoon depicting viral approach to administer a cre-dependent adeno-associated-virus containing excitatory MCH DREADDs-mCherry transgene (AAV2-DIO-rMCHp-hM3D(Gq)-mCherry) into the LHA and ZI. IP DCZ does not activate the DREADD unless Cre is also administered. **b-d** Training data for the Pavlovian Discrimination Task (data were analyzed using a one-way ANOVA with repeated measures and multiple comparisions, n=7 rats) with **b** Average number of licks for sucrose solution per CS+ trial, **c** Average latency to lick from sucrose solution per CS+ trial and **d** Average number of CS+ trials with resonse via licking sucrose solution. **e-f** Effects of IP DCZ during test phase of the Pavlovian Discrimination Task (data analyzed using Students two-tailed paired t-test, n=7 rats) with **e** Average number of licks for sucrose solution per CS+ trial (P=0.9902) and **f** Average latency to lick from sucrose solution per CS+ trial (P=0.8076). Data were analyzed using multiple Student's two-tailed paired t-test. Data shown as mean ± SEM; **P<0.01, ***P<0.001. Source data are provided as a Source Data file. Created with Biorender.com.

Supplementary Figure 2

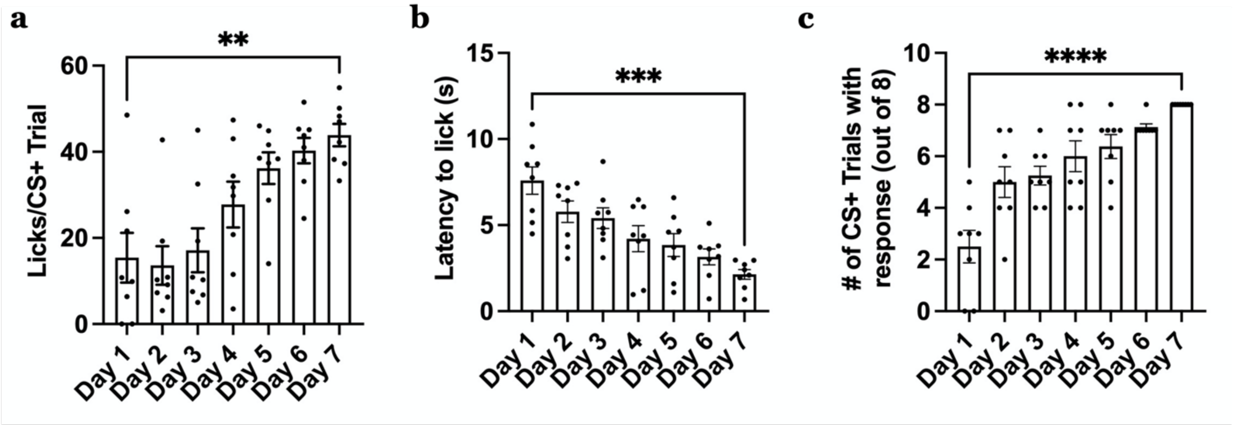

Supplementary Figure 2. Training data for chemogenetic activation of MCH neurons during Pavlovian Discrimination Task in rats (data were analyzed using a one-way ANOVA with repeated measures and multiple comparisions, n=8 rats) with **a** Average number of licks to sucrose solution per CS+ trial, **b** Average latency to lick from sucrose solution per CS+ trial and **c** Average number of CS+ trials with response via licking sucrose solution. Data shown as mean ± SEM; **P<0.01, ***P<0.001, ****P<0.0001. Source data are provided as a Source Data file.

Supplementary Figure 3

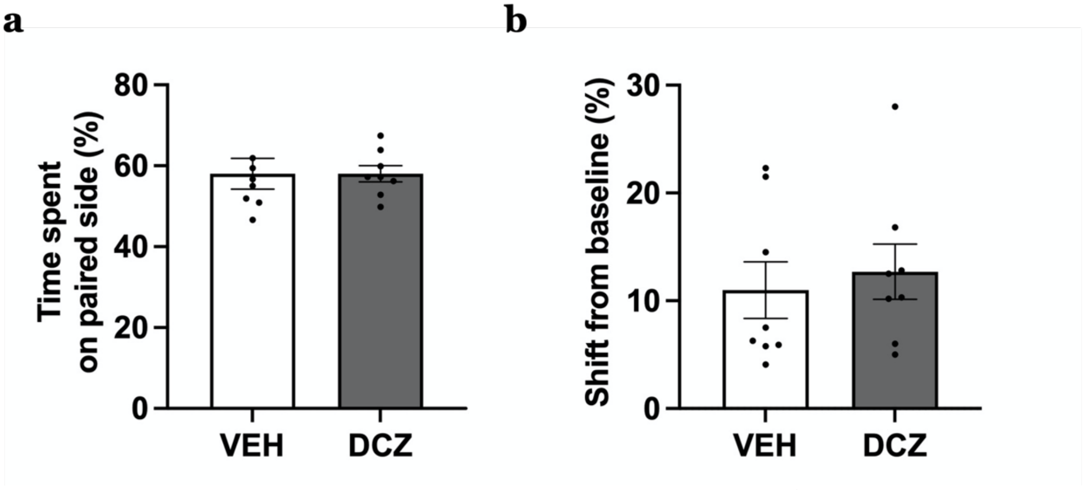

Supplementary Figure 3. IP DCZ does not impact food-seeking memory behavior during CPP in rats (n=7). Animals were injected using a counterbalanced, within subjects design. **a-b** Effects of IP DCZ during test phase of CPP (data analyzed using Students two-tailed paired t-test, n=7 rats) with **a** Time spent on paired side represented as a percentage (P=0.9929) and **b** Shift from baseline (Difference in time spent on side between pre- and post-training, P=0.3684). Data shown as mean ± SEM. Source data are provided as a Source Data file.

Supplementary Figure 4

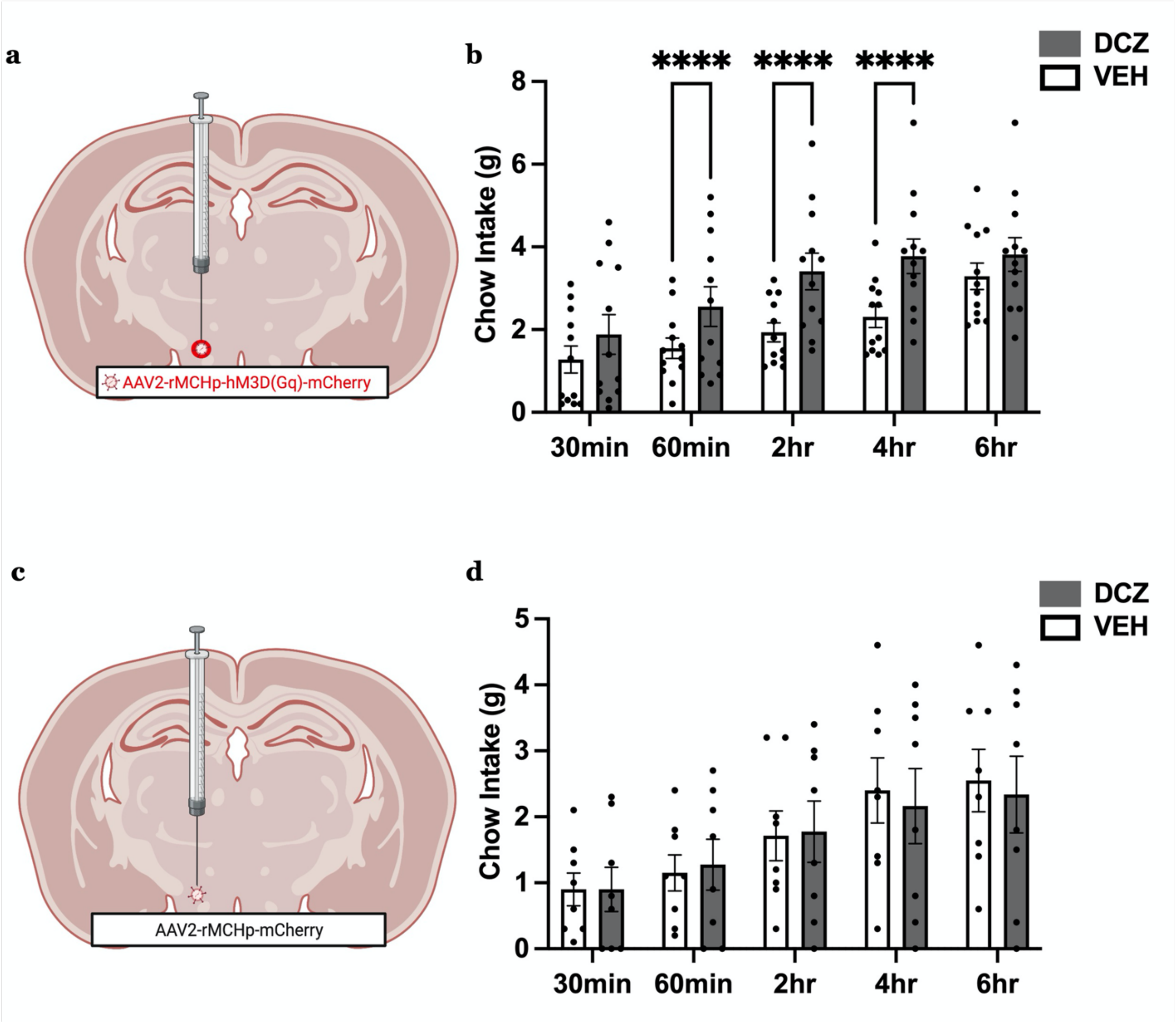

Supplementary Figure 4. Chemogenetic activation of MCH neurons via IP DCZ increases food intake in rats (n=12). Animals were injected using a counterbalanced, within-subject design. **a** Schematic cartoon depicting viral approach to chemogenetically activate MCH neurons. An adeno-associated-virus containing excitatory MCH DREADDs-mCherry transgene (AAV2-rMCHp-hM3D(Gq)-mCherry) is injected into the LHA and ZI. **b** Effect of chemogenetic activation of MCH neurons on home cage chow intake (2hr, **P=0.0027, 4hr, **P =0.0013, n=12 rats). **c** Schematic cartoon depicting viral approach to administer a cre-dependent adeno-associated-virus containing excitatory MCH DREADDs-mCherry transgene (AAV2-DIO-rMCHp-hM3D(Gq)-mCherry) into the LHA and ZI in rats (n=8). **d** Effect of IP DCZ on home cage chow intake (n=8 rats). Data were analyzed using multiple Student's two-tailed paired t-test and ANOVA repeated measures. Data shown as mean ± SEM; ****P<0.0001. Source data are provided as a Source Data file. Created with Biorender.com.

Supplementary Figure 5

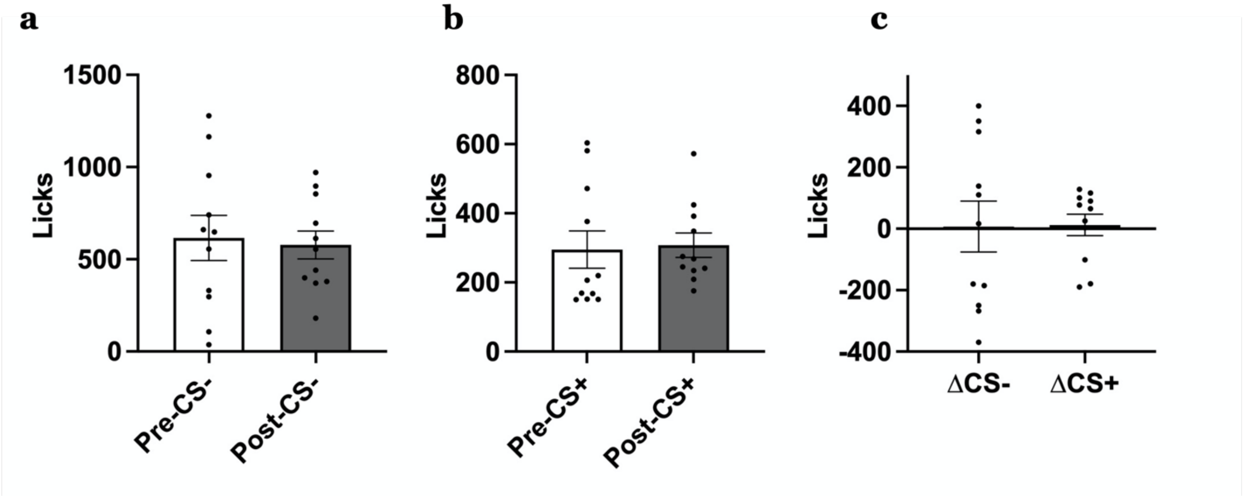

Supplementary Figure 5: IP DCZ does not promote flavor preference conditioning in rats (n=11). Throughout training, animals were counterbalanced and injected using a within-subjects design. **a** Number of licks during the pre- and post-two bottle preference tests for the vehicle-paired CS- (P=0.7952 and **b** DCZ paired CS+ (P=0.7313). **c** Difference in number of licks between pre and post two bottle preference tests for the CS- and CS+ (P=0.9555). Data were analyzed using multiple Student's two-tailed paired t-test. Data shown as mean ± SEM. Source data are provided as a Source Data file.

Supplementary Figure 6

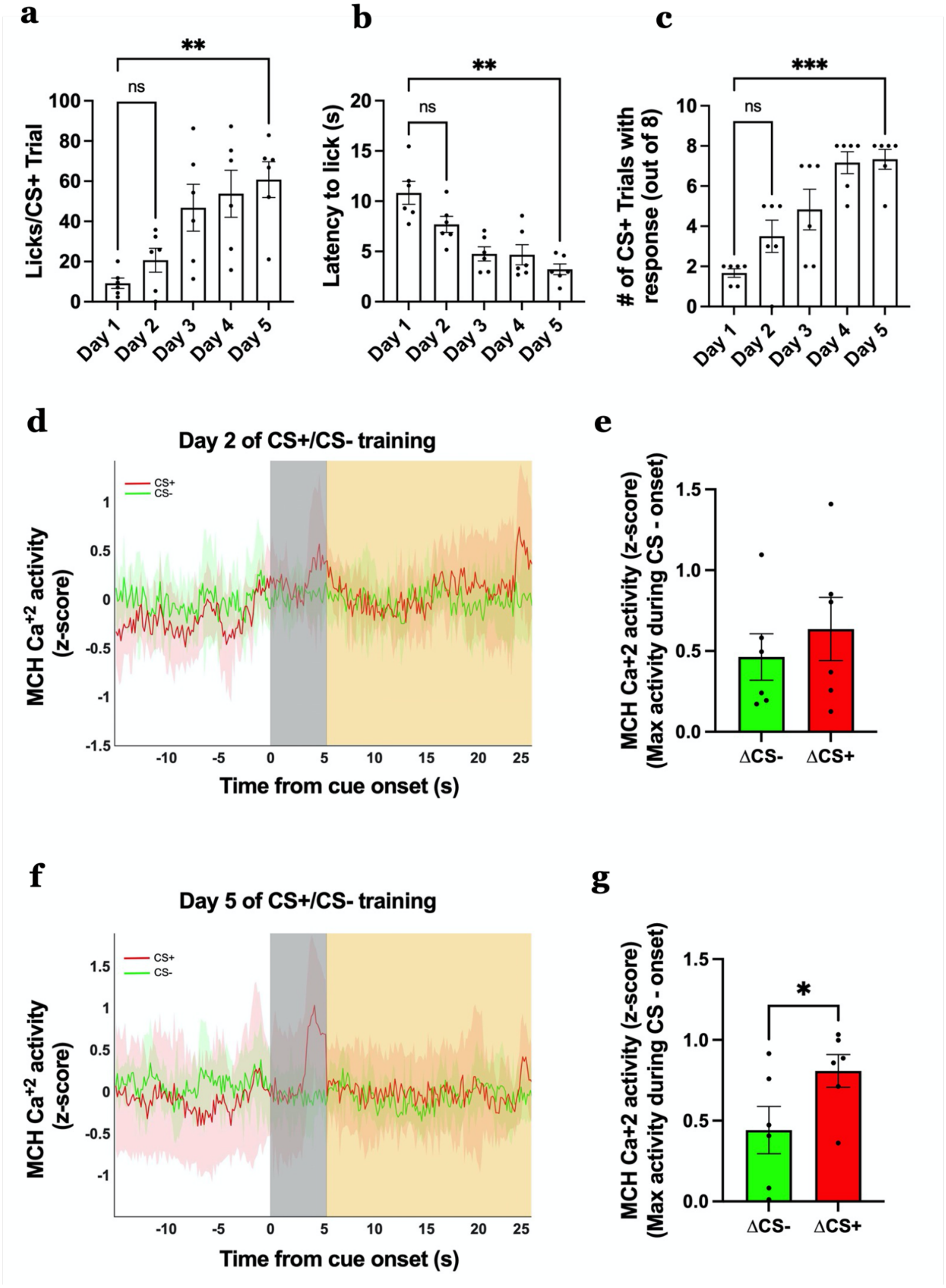

Supplementary Figure 6: Physiological MCH neuron Ca^{2+} activity increases in response to discrete food-predictive cues by the 5th CS training session, thus corresponding with behavioral evidence of learning in rats (n=6). a-c Training data for the first five CS sessions of the Pavlovian Discrimination Task (data were analyzed using a one-way ANOVA with repeated measures and multiple comparisions, n=6 rats): **a** Average number of licks for sucrose solution per CS+ trial, **b** Average latency to lick from sucrose solution per CS+ trial, and **c** Average number of CS+ trials with response via licking sucrose solution. **d-e** Fiber photometry recording of MCH neuron Ca^{2+} activity during the 2nd CS training session (data analyzed using Students two-tailed paired t-test, n=6 rats) **d** Trace of MCH neuron Ca^{2+} activity with MCH neuron Ca^{2+} activity (z-score) time locked to cue onset (CS+ in red and CS- in green; −15 to 25 s relative to the start of the 5s cue [gray box] **e** MCH neuron Ca^{2+} activity during cue period [gray box] (max activity during CS – activity during cue onset, P=0.5757) **f-g** Fiber photometry recording of MCH neuron Ca^{2+} activity during the 5th CS training session (data analyzed using Students two-tailed paired t-test, n=6 rats) **f** Trace of MCH neuron Ca^{2+} activity with MCH neuron Ca^{2+} activity (z-score) time locked to cue onset (CS+ in red and CS- in green; −15 to 25 s relative to the start of the 5s cue [gray box] **g** MCH neuron Ca^{2+} activity during cue period [gray box] (max activity during CS – activity during cue onset, *P=0.0141). **P<0.01, ***P<0.001. Source data are provided as a Source Data file.
