# Supplementary material for: Hypothalamic melanin-concentrating hormone neurons integrate food-motivated appetitive and consummatory processes in rats
Source: Nat Commun. 2023 Mar 29;14:1755. doi: 10.1038/s41467-023-37344-9 (PMC10060386; doi:10.1038/s41467-023-37344-9)
Supplement: Supplementary file 3 — Reporting Summary [file 41467_2023_37344_MOESM3_ESM.pdf]

nature portfolio

Corresponding author(s): Scott E Kanoski

Last updated by author(s): 03/07/2023

Reporting Summary

Nature Portfolio wishes to improve the reproducibility of the work that we publish. This form provides structure for consistency and transparency in reporting. For further information on Nature Portfolio policies, see our Editorial Policies and the Editorial Policy Checklist.

Statistics

For all statistical analyses, confirm that the following items are present in the figure legend, table legend, main text, or Methods section.

n/a	Confirmed	
☐	☒	The exact sample size (*n*) for each experimental group/condition, given as a discrete number and unit of measurement
☐	☒	A statement on whether measurements were taken from distinct samples or whether the same sample was measured repeatedly
☐	☒	The statistical test(s) used AND whether they are one- or two-sided<br>*Only common tests should be described solely by name; describe more complex techniques in the Methods section.*
☒	☐	A description of all covariates tested
☐	☒	A description of any assumptions or corrections, such as tests of normality and adjustment for multiple comparisons
☐	☒	A full description of the statistical parameters including central tendency (e.g. means) or other basic estimates (e.g. regression coefficient) AND variation (e.g. standard deviation) or associated estimates of uncertainty (e.g. confidence intervals)
☐	☒	For null hypothesis testing, the test statistic (e.g. *F*, *t*, *r*) with confidence intervals, effect sizes, degrees of freedom and *P* value noted<br>*Give P values as exact values whenever suitable.*
☒	☐	For Bayesian analysis, information on the choice of priors and Markov chain Monte Carlo settings
☒	☐	For hierarchical and complex designs, identification of the appropriate level for tests and full reporting of outcomes
☒	☐	Estimates of effect sizes (e.g. Cohen's *d*, Pearson's *r*), indicating how they were calculated

Our web collection on statistics for biologists contains articles on many of the points above.

Software and code

Policy information about availability of computer code

Data collection	Custom code was created for data collection using Med- Associates software (Med Associates, Fairfax, VT, USA), which is available upon request and Matlab 2019a. Original Matlab code and a demo file is available in the Open Science Framework Repository https://doi.org/10.17605/OSF.IO/WMKGJ upon the date of publication.
Data analysis	Statistics were performed using GraphPad Prism 9.0 Software (GraphPad Software Inc., San Diego, CA, USA) or Microsoft Excel for Mac (v. 16.66.1; Microsoft Inc., Redmond, WA, USA.

For manuscripts utilizing custom algorithms or software that are central to the research but not yet described in published literature, software must be made available to editors and reviewers. We strongly encourage code deposition in a community repository (e.g. GitHub). See the Nature Portfolio guidelines for submitting code & software for further information.

Data

Policy information about availability of data

All manuscripts must include a data availability statement. This statement should provide the following information, where applicable:

- Accession codes, unique identifiers, or web links for publicly available datasets
- A description of any restrictions on data availability
- For clinical datasets or third party data, please ensure that the statement adheres to our policy

All data generated and analyzed for this manuscript are available from the corresponding senior author (S.E.K.) upon reasonable request. The source data

underlying Figs. 1d–f, h-k,2c-h, 3d-j, 4d–o, i-l, 5c-e, g and 6b-d; Supplemental Figs. 1b-f, 2a-c, 3a-b, 4b,d, 5a-c and 6a-c,e,g are provided as a source datafile with this paper. The data from this manuscript are available in the Open Science Framework Repository https://doi.org/10.17605/OSF.IO/WMKGJ upon the date of publication.

Human research participants

Policy information about studies involving human research participants and Sex and Gender in Research.

Reporting on sex and gender	N/A
Population characteristics	N/A
Recruitment	N/A
Ethics oversight	N/A

Note that full information on the approval of the study protocol must also be provided in the manuscript.

Field-specific reporting

Please select the one below that is the best fit for your research. If you are not sure, read the appropriate sections before making your selection.

☒ Life sciences ☐ Behavioural & social sciences ☐ Ecological, evolutionary & environmental sciences

For a reference copy of the document with all sections, see nature.com/documents/nr-reporting-summary-flat.pdf

Life sciences study design

All studies must disclose on these points even when the disclosure is negative.

Sample size	The number of animals to be used for each proposed experiment is based on power analyses conducted that considered our own pilot work. Power analyses were conducted for each proposed experiment using GraphPad Prism 9.0 Software (GraphPad Software Inc., San Diego, CA, USA) or Microsoft Excel for Mac (v. 16.66.1; Microsoft Inc., Redmond, WA, USA. Alpha level was set at 0.05 for power analyses. The group sizes were chosen based on the minimum number of animals to provide statistical power to achieve detection of statistically significant differences based on the power analyses described above.
Data exclusions	Animals were excluded based on the following pre-determined criteria: 1) when the animals lost signal or did not have a successful recording session during behavior (including Pavlovian Discrimination Task, CPP and refeeding, n=2) 2) where they failed respond to the CS+ during Pavlovian Discrimination Conditioning or PIT (n=2 for all experiments) 3) where they failed to meet the nimum criterion of mCherry positive cells in DREADDs experiments (n=1) 4) where they were determined to be statistically significant outliers based on the Grubbs test using an alpha level of .05 (n=1 for all experiments) .
Replication	For immunohistological colocalization studies with the AAV9.pMCH.GCaMP6s.hGH (Figure 1b) data was replicated in n=6 rats and with the AAV2.rMCHp.hM3D(Gq).mCherry virus (Figure 4b) were replicated in n=8 rats.
Randomization	Though most of the experiments utilized a within-subjects design, the order of treatments was counterbalanced based on initial body weight where behavioral data (licks, lever presses, %time spent in context, etc) was not relevant to the experiment and based on behavioral activity (licks, lever presses, % time spent in context, etc) prior to test day for tasks in the operant chambers
Blinding	Investigators running behavioral experiments, and weighing food intake and body weights were blinded to treatment groups during data collection and data analysis.

Reporting for specific materials, systems and methods

We require information from authors about some types of materials, experimental systems and methods used in many studies. Here, indicate whether each material, system or method listed is relevant to your study. If you are not sure if a list item applies to your research, read the appropriate section before selecting a response.

Materials & experimental systems

n/a	Involved in the study	
☐	☒	Antibodies
☒	☐	Eukaryotic cell lines
☒	☐	Palaeontology and archaeology
☐	☒	Animals and other organisms
☒	☐	Clinical data
☒	☐	Dual use research of concern

Methods

n/a	Involved in the study	
☒	☐	ChIP-seq
☒	☐	Flow cytometry
☒	☐	MRI-based neuroimaging

Antibodies

Antibodies used	Rabbit anti-MCH (1:5000; PhoenixPharmaceuticals, Burlingame, CA, USA; Catalog #: H-070-47; Clonality: Polyclonal; Lot #: 46317), and rabbit anti-RFP (1:2000, Rockland Inc.,Limerick, PA, USA; Catalog #:600-401-379; Clonality: Polyclonal) were the two antibodies used. The two secondary antibodies used , donkey anti-rabbit AF647(Catalog #: 711-606-152; Lot #: 160172) and donkey anti-rabbit AF488 (Catalog #: 711-546-152; Lot #: 126798) had a 1:500 dilution and stored overnight at 4 °C (Jackson Immunoresearch; West Grove, PA, USA).
Validation	The MCH primary antibody was validated based on comparisons for MCH positive signal colocalized with mCherry immunofluorescence in animals expressing the MCH-mCherry transgene (delivered via virogenetics). In addition, prior reports using this same antibody have shown a lack of MCH immunohistochemical staining using the antibody when MCH neurons are ablated (PMID 23365238), further validating the primary is specific to MCH neurons. RFP antibody showed no immunoreactivity in non-injected animals, confirming the specificity of RFP to mCherry (virogenetically delivered to MCH neurons).

Animals and other research organisms

Policy information about studies involving animals; ARRIVE guidelines recommended for reporting animal research, and Sex and Gender in Research

Laboratory animals	Adult Male Sprague Dawley rats (Envigo, Indianapolis, IN, USA) weighing 300-400g were used for this study.
Wild animals	No wild animals were used in this study
Reporting on sex	Only male rats were used in this study.
Field-collected samples	No field-collected samples were used in this study
Ethics oversight	Experiments were performed in accordance with NIH Guidelines for the Care and Use of Laboratory Animals, and all procedures were approved by the Institutional Animal Care and Use Committee of the University of Southern California.

Note that full information on the approval of the study protocol must also be provided in the manuscript.
