# Supplementary material for: Hypothalamic melanin-concentrating hormone neurons integrate food-motivated appetitive and consummatory processes in rats
Source: Nat Commun. 2023 Mar 29;14:1755. doi: 10.1038/s41467-023-37344-9 (PMC10060386; doi:10.1038/s41467-023-37344-9)
Supplement: Supplementary file 4 — Source Data [file 41467_2023_37344_MOESM4_ESM.pdf]

Figure 1d

Rat ID	Day 1	Day 2	Day 3	Day 4	Day 5
B1801	9.625	0	40.25	87.25	82.875
B1802	2.25	25.875	68.25	75.25	71.25
B1803	7.125	32.375	86.25	70.25	52.125
B1804	4.375	24.75	54.25	15.75	21.125
B1805	20.125	35.75	11.375	26.25	66.75
B1806	11.75	5.375	20.25	47.75	70.625

Day 6	Day 7
72.5	80.125
59.5	49
71.625	75
52.625	48.5
56.25	55.75
25.25	43.75

Figure 1e

Rat ID	Day 1	Day 2	Day 3	Day 4	Day 5
B1801	7.725	6.7	4.2875	3.32	1.32375
B1802	12.03	5.183333	4.908571	2.695	2.60875
B1803	8.91	10.934	2.917143	3.62	2.972857
B1804	15.465	8.596667	2.971429	2.89125	2.9775
B1805	9.29	6.81	6.41	8.558	4.588
B1806	11.565	7.91	7.07	6.973333	4.87625

Day 6	Day 7
0.67	0.9525
1.2675	0.807143
1.28125	0.564286
0.67875	0.6675
2.071429	1.468571
4.34375	2.79875

Figure 1f

Rat ID	Day 1	Day 2	Day 3	Day 4	Day 5
B1801	2	0	4	8	8
B1802	1	3	7	8	8
B1803	1	5	7	8	7
B1804	2	3	7	8	8
B1805	2	5	2	5	5
B1806	2	5	2	6	8

Day 6	Day 7
8	8
8	8
8	8
8	8
7	7
8	8

Figure 1h

	CS-				
	1	2	3	4	5
B1801	-0.0167	0.004	-0.0235	0.1173	0.1994
B1802	-0.3814	-0.3151	-0.3579	-0.3474	-0.1927
B1803	-0.2313	-0.2833	-0.1745	-0.2666	-0.0796
B1804	-0.0337	0.0398	0.0935	0.204	0.1896
B1805	-0.0173	0.0242	-0.3118	-0.2897	-0.3735
B1806	0.123	-0.0744	0.0563	0.1324	-0.241

CS+				
1	2	3	4	5
0.2371	0.4238	0.3577	0.6487	0.8884
0.1473	0.2692	0.5977	0.6719	0.8146
0.3566	0.3118	0.5196	0.5826	0.7098
0.197	0.3794	0.6119	0.7916	1.2296
0.1443	0.3754	0.5364	0.6291	0.7939
0.2707	0.3273	0.339	0.6785	0.9289

Figure 1i

Rat ID	ΔCS-	ΔCS+
B1801	0.2957	0.7765
B1802	0.1771	0.8033
B1803	0.4486	0.7497
B1804	0.6403	1.7208
B1805	0.6294	1.0246
B1806	0.6777	1.4163

Figure 1j

	CS-				
	B1801	B1802	B1803	B1804	B1805
5s-pre-CS	0.0723	0.0481	0.0153	-0.0065	-0.1415
5s-CS period	0.0647	-0.3165	-0.1959	0.0705	-0.2058
5s-post-CS	0.1102	0.3862	-0.0197	0.07	-0.2018

	CS+						
B1806	B1801	B1802	B1803	B1804	B1805	B1806	
0.127	-0.0318	0.125	0.17	0.1916	0.3311	-0.055	
0.0054	0.5164	0.4984	0.4983	0.6426	0.4912	0.5119	
0.0132	0.0987	0.2161	-0.3154	-0.1416	-0.0377	0.0038	

Figure 1k

Rat ID	Trial #	Latency	ΔCS+
B1801	1	0.69	1.956763
B1801	2	2.87	-0.042137
B1801	3	0.7	1.432763
B1801	4	0.82	0.510063
B1801	5	0.75	0.736063
B1801	6	0.78	0.550363
B1801	7	0.99	0.521563
B1801	8	0.887	0.762363
B1802	1	0.7	0.693263
B1802	2	0.61	1.570563
B1802	3	0.78	0.761963
B1802	4	1.13	0.247163
B1802	5	0.64	1.328763
B1802	6	4.5	-1.123237
B1802	7	0.78	1.070763
B1802	8	7.39	-1.113187
B1803	1	2.3	-0.133537
B1803	2	5.63	-1.111777
B1803	3	2.28	-0.167237
B1803	4	0.85	0.851763
B1803	5	2.12	-0.259437
B1803	6	2.14	-0.268637
B1803	7	2.26	-0.256837
B1803	8	0.7	0.780163
B1804	1	0.71	1.596763
B1804	2	0.73	1.371963
B1804	3	0.84	0.482863
B1804	4	0.55	1.641163
B1804	5	5.91	-1.104347
B1804	6	0.84	0.722763
B1804	7	0.83	0.503463
B1804	8	0.69	1.098963
B1805	1	5.87	-0.925137
B1805	2	1	-0.167837
B1805	3	0.74	0.461463
B1805	4	0.73	1.204463
B1805	5	0.61	1.395463
B1805	6	0.73	1.048163
B1805	7	0.73	0.444063
B1806	1	0.97	-0.230237
B1806	2	0.94	-0.298537

B1806	3	1.31	-0.270437
B1806	4	0.75	1.688863
B1806	5	0.75	1.219563
B1806	6	0.72	1.443963
B1806	7	0.77	1.567263
B1806	8	7.04	-1.097237

Figure 2c

Rat ID	unpaired (%)	paired (%)
8408	28.9	71.1
9012	42	58
9014	40.4	59.6
9301	43.3	56.7
9302	38.6	61.4
9308	35.8	64.2

Figure 2d

Rat ID	unpaired (%)	paired (%)
8408	-34.2	34.2
9012	-14.6	14.6
9014	-19.6	19.6
9301	-12.6	12.6
9302	-18.7	18.7
9308	-17.1	17.1

Figure 2e

Rat ID	unpaired	paired
8408	0.16041429	0.39442857
9012	0.11092	0.2204625
9014	0.023035	0.20855789
9301	0.01475714	0.4100375
9302	-0.2317167	0.4266
9308	-0.1772912	0.51205714

Figure 2f

Rat ID	unpaired	paired
8408	-0.2577143	0.69848571
9012	-0.18542	0.4962875
9014	-0.327015	0.25154211
9301	-0.3469571	0.4125125
9302	-0.2543333	0.3021
9308	-0.4686143	0.10224286

Figure 2g

Rat ID	Activity upon entry to paired side	Shift from baseline
8408	0.69848571	34.2
9012	0.10224286	14.6
9014	0.4962875	19.6
9301	0.3021	12.6
9302	0.4125125	18.7
9308	0.25154211	17.1

Figure 2h

Rat ID	Activity in paired side	Shift from baseline
8408	0.51205714	34.2
9012	0.2204625	14.6
9014	0.20855789	19.6
9301	0.4100375	12.6
9302	0.4266	18.7
9308	0.39442857	17.1

Figure 3d

Rat ID	Within Bouts	Interbouts
8408	2.37852	-0.86315
8710	1.302175	-0.423233
9012	0.5574	-0.18125
9014	0.74771111	-0.536188
9301	2.030345	-1.10238
9302	0.75822	-0.549475
9303	0.5231625	-0.633371

Figure 3e

Rat ID	Within bouts	Interbouts
8408	2.37852	-0.86315
8710	1.302175	-0.423233
9012	0.5574	-0.18125
9014	0.74771111	-0.536188
9301	2.030345	-1.10238
9302	0.75822	-0.549475
9303	0.5231625	-0.633371

Figure 3f

Rat ID	Pre-food access	Post-last bout
8408	-1.238	1.83
8710	-0.9839	5.8
9012	1.359	3.275
9014	-1.398	6.32
9301	-3.039	-0.5244
9302	-1.554	2.281
9303	-0.7628	0.4429

Figure 3g

Rat ID	Chow intake	Within bouts
8408	5.2	2.37852
8710	4.1	1.302175
9012	3.1	0.5574
9014	3.6	0.74771111
9301	4.8	2.030345
9302	3.56	0.75822
9303	2.6	0.5231625

Figure 3h

Rat ID	Chow intak	ΔAUC
8408	5.2	7.718
8710	4.1	2.5146
9012	3.1	1.916
9014	3.6	3.068
9301	4.8	6.7839
9302	3.56	3.835
9303	2.6	1.2057

Figure 3i

Rat ID	Time Point	Within Bout activity
A8408	1	3.1655
A8408	1	2.908
A8408	2	1.7817
A8408	3	2.5704
A8408	3	1.467
A8710	1	2.6319
A8710	2	0.4451
A8710	2	1.4588
A8710	3	0.6729
A9012	1	1.0646
A9012	1	1.0351
A9012	2	-0.4671
A9012	3	0.4911
A9012	3	0.6633
A9014	1	1.1121
A9014	1	1.989
A9014	1	0.556
A9014	2	1.5841
A9014	2	-0.1997
A9014	2	1.531
A9014	3	-0.4467
A9014	3	1.1832
A9014	3	-0.5796
A9301	1	2.92847
A9301	1	3.3694
A9301	2	2.8495
A9301	2	1.9064
A9301	3	1.7608
A9301	3	-0.6325
A9302	1	1.2038
A9302	1	1.2627
A9302	2	0.5256
A9302	3	0.4186
A9302	3	0.3804
A9303	1	1.1716
A9303	1	0.3836
A9303	1	0.3415
A9303	2	0.781
A9303	2	1.0431
A9303	3	0.0993
A9303	3	0.1482

A9303	3	0.4032

Figure 3j

Rat ID	bout length	Within Bouts
A8408	1.5808	-0.91935
A8408	0.6223	-1.119967
A8408	2.2508	-0.5072
A8408	1.7383	-0.198475
A8408	3.0213	0.294334
A8710	5.906513	2.6319
A8710	0.75437	0.4451
A8710	6.718318	1.4588
A8710	4.514177	0.6729
A9012	3.8514	1.0646
A9012	1.8343	0.6633
A9012	1.2918	-0.4671
A9012	0.8069	0.4911
A9012	4.8668	1.0351
A9014	0.5008	1.1121
A9014	0.593	-0.4467
A9014	2.8362	0.556
A9014	1.3378	1.5841
A9014	2.0918	-0.1997
A9014	6.9095	1.531
A9014	5.9306	1.989
A9014	4.7856	1.1832
A9014	0.7749	-0.5796
A9301	4.0556	1.7608
A9301	5.0829	-0.6325
A9301	0.756	3.3694
A9301	0.6224	1.9064
A9301	7.6073	2.8495
A9301	5.8082	2.92847
A9302	4.7765	0.4186
A9302	4.1876	1.2627
A9302	3.2178	0.5256
A9302	1.702	1.2038
A9302	3.5456	0.3804
A9303	3.529	0.3415
A9303	0.8677	0.3836
A9303	0.6504	0.1482
A9303	3.861	0.781
A9303	1.4762	1.0431
A9303	1.8111	0.0993
A9303	1.5102	1.1716

A9303	0.922	0.4032

Figure 4d

Rat ID	Day 1	Day 2	Day 3	Day 4	Day 5
4802	9.125	56.875	66	61.1	77.625
4803	31.1	37.25	37.7	51.1	52.25
4804	53.75	63.25	75.125	51.375	89.375
4805	9.875	12.375	9.375	14.5	9.25
4806	27.5	30.875	27.625	23.625	33.3
4807	10.1	10.5	33.3	31.1	34.4
4808	14	38.5	57.75	55	61.25
4809	8.125	8.625	9.625	8.125	15.125
4810	60.75	56.875	52.625	60.875	53.625
4811	28.125	39.375	38.125	44.25	58.375
4812	31.25	29.625	40.625	48.25	68.625
4813	20.875	24	19.125	33	43.375

Day 6	Day 7
75.5	76.625
51.75	61
87.7	88.75
9.9	24.5
22.75	33.12
24.875	42.625
58.125	57.875
15.5	22.125
77.625	75.125
47.125	52.12
61.875	65.5
43	46.5

Figure 4e

Rat ID	Day 1	Day 2	Day 3	Day 4	Day 5
4802	2.68	6.15625	2.22	2.225	1.51
4803	1.1875	2.5775	1.365	0.76	0.675
4804	5.307143	5.885	0.95875	0.85625	2.0625
4805	1.998333	2.098	8.271429	3.958333	6.343333
4806	5.7725	5.38125	2.0425	0.825	2.0325
4807	10.19	0.68	5.216667	0.77	0.504286
4808	9.155	5.144286	3.652857	2.65	1.115
4809	6.876667	10.21	6.04	8.6275	3.85375
4810	4.354286	5.736	2.183333	2.022857	1.8575
4811	6.028333	3.72125	0.94	1.15375	0.9025
4812	1.734286	1.37125	3.69125	0.68625	0.85625
4813	2.41125	0.8475	1.4375	0.65125	0.68125

Day 6	Day 7
0.96875	0.85125
0.8125	0.90875
0.875	0.7975
7.45	4.99
2.8925	2.4425
1.26375	0.69375
0.6525	1.04
4.12	3.45
0.83625	0.69125
6.41125	0.74875
0.70125	0.69
0.71	0.79625

Figure 4f

Rat ID	Day 1	Day 2	Day 3	Day 4	Day 5
4802	1	8	8	8	8
4803	8	8	8	8	8
4804	7	8	8	8	8
4805	6	5	7	6	6
4806	4	8	8	8	8
4807	1	1	6	8	7
4808	2	7	8	7	8
4809	3	4	7	8	8
4810	7	5	7	7	8
4811	6	8	8	8	8
4812	7	8	8	8	8
4813	8	8	8	8	8

Day 6	Day 7
8	8
8	8
8	8
5	8
8	8
8	8
8	8
8	8
8	8
8	8
8	8
8	8

Figure 4g

Rat ID	Vehicle	DCZ
5804	45.2	58.3
5807	39	43.5
5808	39.4	49.4
5809	24.4	44.4
5810	33	44.625
5811	46.125	55.125
5812	67.44	68.4
5813	58	65.4

Figure 4h

Rat ID	Vehicle	DCZ
5804	3.045	2.40125
5807	2.51125	3.23
5808	2.1475	1.3675
5809	3.65	0.6383
5810	2.992	0.741429
5811	1.35375	0.77
5812	2.975	2.3222
5813	3.94	1.433

Figure 4i

Rat ID	Day 1	Day 2	Day 3	Day 4
4802	1.875	4.375	1.125	0.875
4803	56.875	48.75	54.75	66.875
4804	3.875	17.125	1.625	3.125
4805	2.5	10.625	6.25	9.375
4806	18.875	4.5	21.875	16.25
4807	10.125	2.875	2.375	1.625
4808	47.5	3.875	42.25	48.625
4809	2.875	12.375	6.75	5.625
4810	5.125	25.25	16.25	63.625
4811	40.625	10.5	12.625	27
4812	10.5	12.375	12.25	7.25
4813	16.75	29.625	4.125	6.625

Day 5	Day 6	Day 7	Day 8	Day 9	Day 10	Day 11
6.5	26.25	69.75	67.625	72.375	75	72.375
20.75	42.625	36	19	32.125	59.5	52
6.375	7.25	30.5	41.875	70.25	74.25	71.625
18.75	26.25	18.75	22.5	35	37.25	37.25
11.125	1.875	60.75	67.75	62.75	63.375	75.125
10	5	16.125	14.5	29.25	24	33.125
36	31.875	30.25	24.5	25.75	9.875	21.875
22.5	25	15.625	31.25	39.25	39	39.375
38.625	12.125	77	64.75	60	50.25	52.125
29.25	6.25	38.75	18.875	19.625	13.125	10.625
33.625	10.875	34.625	28.875	36.125	15.875	20.625
29	10.625	6.125	3.625	7	6.375	6.125

Day 12
69.5
42.75
75.625
51.5
60.5
51.125
33
51.375
39.25
13.375
48.5
22.75

Figure 4j

Rat ID	Day 1	Day 2	Day 3	Day 4
4802	75	50	25	25
4803	87.5	100	100	100
4804	37.5	37.5	50	37.5
4805	12.5	25	37.5	62.5
4806	75	87.5	50	50
4807	62.5	37.5	37.5	37.5
4808	87.5	100	87.5	100
4809	12.5	25	50	62.5
4810	100	100	75	87.5
4811	100	100	100	100
4812	75	100	75	75
4813	75	75	100	100

Instrumental Conditioning

Day 5	Day 6	Day 7	Day 8	Day 9	Day 10	Day 11
50	50	100	100	100	100	100
100	75	100	100	100	100	100
37.5	37.5	50	62.5	100	100	100
62.5	75	75	75	87.5	100	100
37.5	25	100	100	100	100	100
50	25	25	25	50	87.5	100
87.5	62.5	100	100	100	100	100
62.5	75	87.5	75	87.5	87.5	100
62.5	100	100	100	100	100	100
87.5	100	100	100	100	100	100
62.5	100	75	100	100	100	100
100	87.5	100	100	100	100	100

Instrur

Day 12	Rat ID	Day 1	Day 2	Day 3	Day 4	Day 5
100	4802	87.5	75	50	37.5	31.25
100	4803	100	87.5	100	37.5	62.5
100	4804	87.5	100	75	50	43.75
100	4805	12.5	0	12.5	0	6.25
100	4806	100	87.5	75	62.5	68.75
100	4807	87.5	87.5	62.5	62.5	56.25
100	4808	100	100	87.5	50	87.5
100	4809	12.5	0	25	25	18.75
100	4810	100	87.5	100	62.5	75
100	4811	100	100	50	75	75
100	4812	100	100	100	87.5	62.5
100	4813	100	100	100	87.5	81.25

nental Extinction

Day 6	Day 7	Day 8	Day 9	Day 10	Day 11	Day 12
31.25	25	0	12.5	25	50	25
62.5	68.75	43.75	37.5	25	25	12.5
43.75	43.75	62.5	50	31.25	12.5	12.5
0	0	0	0	6.25	0	6.25
50	43.75	50	25	18.75	25	31.25
25	50	50	43.75	18.75	25	25
87.5	62.5	37.5	31.25	43.75	31.25	18.75
12.5	12.5	6.25	6.25	18.75	25	0
62.5	62.5	43.75	56.25	75	50	31.25
31.25	50	50	37.5	37.5	25	18.75
62.5	37.5	81.25	37.5	31.25	31.25	18.75
93.75	81.25	50	62.5	43.75	25	25

Figure 4k

Rat ID	Day 1	Day 2	Day 3	Day 4
4802	8.296667	11.88	4.315	13.345
4803	2.848571	1.92125	1.52125	0.8825
4804	8.993333	5.13	3.95	23.15
4805	12.4	8.1	11	6.7
4806	11.51333	6.028571	10.76	9.3375
4807	10.274	10.29	1.863333	2.496667
4808	8.037143	14.74875	4.387143	2.14625
4809	14.5	15	12.8	8.5
4810	5.96375	10.4125	8.828333	6.11
4811	9.93875	6.18125	8.28625	4.30125
4812	4.56	8.725	14.48833	12.62167
4813	2.536667	2.015	9.3275	5.9025

Instrumental Conditioning

Day 5	Day 6	Day 7	Day 8	Day 9	Day 10	Day 11
10.305	1.47	12.70375	2.05	3.6575	2.72	1.34125
0.97375	2.116667	0.7375	1.925	0.5275	0.48	0.6575
14.49	14.6	15.29	16.842	2.6975	2.555	2.55875
6.7	12	7.4	6.1	4	2.8	2.1
6.636667	8.295	7.0575	4.12875	1.795	2.99875	2.13
12.64	12.14	10.22	16.005	16.07	10.92714	7.9275
8.797143	7.58	2.82125	2.2525	3.94	1.505	1.3825
8.8	13.2	6.4	6.3	3.7	3.4	1.9
6.306	3.8	1.60375	3.0525	3.38875	3.175	1.36
5.507143	4.87125	5.28375	3.61	3.1475	2.39375	2.3275
11.98	1.78125	8.688333	2.18125	3.70875	1.095	0.88
6.5325	2.805714	2.4375	1.1075	1.15875	1.39125	1.0575

Instrur

Day 12	Rat ID	Day 1	Day 2	Day 3	Day 4	Day 5
1.477143	4802	1.838333	18.3175	5.516667	13.296	5.486
0.745	4803	0.917143	2.2875	5.066667	4.401	6.208
1.09	4804	3.62375	1.911667	9.8375	6.687143	6.258571
1.5	4805		15		17.55	
1.138571	4806	4.338571	4.028333	4.272	6.146364	5.68
3.774286	4807	2.737143	6.158	1.02	5.473333	10.4275
0.895	4808	3.50125	2.805714	3.535	5.398571	11.73071
1.6	4809		17.645	13.785	11.05	23.595
1.468571	4810	3.698571	2.45375	7.896	8.415833	8.897
2.53125	4811	5.525	4.0925	4.181667	12.555	5.476
2.25125	4812	1.36875	3.07625	3.712857	4.055	6.045
0.89	4813	1.19	0.96125	1.427143	5.591538	3.732

nental Extinction

Day 6	Day 7	Day 8	Day 9	Day 10	Day 11	Day 12
7.235		17.44	12.8225	12.177	13.68	25
4.201818	7.115714	4.878333	1.2975	9.2975	4.16	12.5
13.78	8.585	11.38625	3.026	4.81	5.255	12.5
			6.33		7.67	6.25
9.214286	8.55375	2.8075	2.633333	6.1525	9.198	31.25
8.4075	7.39875	9.517143	18.50333	15.025	24.505	25
8.081	7.341667	5.788	9.722857	3.836	14.13667	18.75
11.63	5.49	3.44	11.95667	3.035		0
9.544	4.731429	6.232222	7.165833	9.29875	7.248	31.25
9.2175	5.95875	3.073333	6.921667	11.4825	20.04667	18.75
7.536667	7.178462	2.66	10.75	6.434	9.753333	18.75
8.170769	8.07875	10.549	9.18	2.5925	12.4025	25

Figure 4l

Rat ID	Vehicle	DCZ
4802	17.2	8.8
4803	10.47	3.12
4804	3.463333	6.5025
4805	6.5025	7.8
4806	12	5.825
4807	2.1075	7.318
4808	13.725	6.96
4809	6.1	3.97
4810	16.962	3.235
4811	16.505	10.5275
4812	10.09	10.035
4813	4.806667	4.02

Figure 4m

Rat ID	Vehicle	DCZ
4802	14.4	10.3
4803	10.55	9.7
4804	7	12.3
4805	8.7	8.7
4806	19.03333	7.5
4807	12.9	6.2
4808	11.4	23.3
4809	16.65	16.65
4810	12.2	12.2
4811	12.3	10.1
4812	7.8	7.8
4813	18.76667	18.76667

Figure 4n

Rat ID	Vehicle	DCZ
4802	1	1
4803	3	2
4804	5	5
4805	1	2
4806	1	5
4807	4	6
4808	2	3
4809	1	3
4810	5	4
4811	2	4
4812	3	4
4813	3	4

Figure 40

Rat ID	Vehicle	DCZ	
4802	2	1	
4803	2	1	s
4804	1	1	
4805	1	1	
4806	3	2	
4807	1	1	
4808	1	1	
4809	2	2	
4810	1	1	
4811	2	1	
4812	2	2	
4813	3	3	

Figure 5b

Rat ID	Vehicle	DCZ
5801	52.8	59
5802	55.8	81.9
5803	53	76.6
5804	51.8	60.6
5805	61.9	60.4
5807	53.3	63.3
5808	63.8	83
5809	60.1	60.6
5810	71.6	75.8
5811	66	66.7
5812	45.1	63.7
5813	60.1	67.9

Figure 5c

Rat ID	Vehicle	DCZ
5801	10	16.2
5802	7.1	33.2
5803	9.6	33.2
5804	5.2	13.9
5805	14	12.4
5807	5.8	15.8
5808	15	34.2
5809	18.2	18.7
5810	24.3	28.6
5811	35.2	35.9
5812	1.6	20.2
5813	13.1	20.9

Figure 5e

Rat ID	Vehicle	DCZ
5801	3.888	4.323
5802	5.258	2.88
5803	6.511	5.327
5804	4.246	6.897
5805	6.526	5.239
5807	6.286	5.419
5808	6.759	7.948
5809	3.757	4.552
5810	3.577	4.938
5811	5.799	5.368
5812	5.553	5.068
5813	7.989	8.296

Figure 5g

	Vehicle				
Rat ID	504	505	506	507	510
Chow Intake	5	3.6	4.3	4.8	7.9

	DCZ					
512	501	502	503	508	509	511
5.5	6.1	5.7	7.7	9	6.7	8.3

Figure 6b

Rat ID	Pre-CS-	Post-CS-
A3702	195	747.5
A3703	1205	1259.5
A3704	1387	738
A3705	515	1209
A3706	1874.5	1044
A3707	433.5	1797.5
A3709	526.5	151
A3710	1122	1634
A3711	843.5	708
A3714	567	418
A3715	342	2179.5
A3716	288.5	448

Figure 6c

Rat ID	Pre-CS+	Post-CS+
A3702	92	1401.5
A3703	1152	2193
A3704	1436.5	558.5
A3705	131.5	2179
A3706	924.5	1545.5
A3707	49.5	722
A3709	255	3874
A3710	112.5	1266.5
A3711	801	1877
A3714	370.5	1404
A3715	108	684.5
A3716	276.5	2065.5

Figure 6d

Rat ID	ΔCS-	ΔCS+
A3702	552.5	1309.5
A3703	54.5	1041
A3704	-649	-878
A3705	694	2047.5
A3706	-830.5	621
A3707	1364	672.5
A3709	-375.5	3619
A3710	512	1154
A3711	-135.5	1076
A3714	-149	1033.5
A3715	1837.5	576.5
A3716	159.5	1789

Supplemental Figure 1b

Rat ID	Day 1	Day 2	Day 3	Day 4
9901	33.625	33.625	41.625	37.5
9902	25.375	20.125	10.875	28.125
9905	13	30.125	23.875	31.375
9906	6.5	11.625	5.125	12.5
9907	19.5	13	13.625	18.125
9908	12.25	25.125	27.375	30.25
9909	16.875	20.375	12.75	18.875

Day 5	Day 6	Day 7
43.375	49.875	52.75
37.25	44.375	48.5
34.625	41.625	47.125
23.375	37.25	44.375
20.75	30.5	36
34.5	40.25	42.875
22.25	31.875	38

Supplemental Figure 1c

Rat ID	Day 1	Day 2	Day 3	Day 4
9901	10.25	1.978	0.9025	1.29625
9902	4.704	2.068	7.481429	5.87125
9905	10.34	5.45875	7.77625	4.90125
9906	10.71	8.9125	11.826	8.455
9907	2.886	3.63	6.12	4.257143
9908	9.345	5.352	4.426	4.988333
9909	8.175	9.632333	2.442857	3.35

Day 5	Day 6	Day 7
1.155	1.1275	1.615
5.921429	4.4	4.10125
6.2475	6.25125	5.86375
8.755	7.13	6.25375
4.437833	6.46	4.4625
5.421667	3.17875	4.426
3.068333	4.414286	2.442857

Supplemental Figure 1d

Rat ID	Day 1	Day 2	Day 3	Day 4
9901	6	5	8	8
9902	5	5	7	8
9905	2	8	8	8
9906	1	4	5	6
9907	5	3	5	7
9908	2	5	5	6
9909	2	6	7	6

Day 5	Day 6	Day 7
8	8	8
7	8	8
8	8	8
6	8	8
6	7	8
6	8	8
6	7	8

Supplemental Figure 1e

Rat ID	Vehicle	DCZ
9901	33.124	33.222
9902	32.875	28
9905	31.25	36.666
9906	36.365	33
9907	36.875	37.55556
9908	34	39.1
9909	38.25	35.333

Supplemental Figure 1f

Rat ID	Vehicle	DCZ
9901	2.89	2.19125
9902	2.98125	2.405
9905	4.211429	7.2
9906	6	5.5
9907	5.35125	3.125
9908	4.0375	4.9
9909	4.11375	3.14875

Supplemental Figure 2a

Rat ID	Day 1	Day 2	Day 3	Day 4
5804	10.75	9.125	5	29.75
5807	9.875	3.125	10.875	47.375
5808	48.5	19.25	19.25	11.5
5809	6.375	10.875	7.5	20.75
5810	0	10.25	10.25	34.375
5811	0	42.75	45	43
5812	26.125	7.25	32.5	31.75
5813	21.625	6.25	6.75	3.5

Day 5	Day 6	Day 7
37.375	38	37.25
41.625	51.5	50.1
14	24.5	33.3
38.375	43.75	42.2
46	45.25	54.875
38.5	41	48.25
44.875	45.125	47
28.75	33.125	38.2

Supplemental Figure 2b

Rat ID	Day 1	Day 2	Day 3	Day 4
5804	10.85667	6.414	5.7	4.38
5807	5.145	7.425	4.52	6.487143
5808	8.526667	3.061429	5.748	0.974
5809	5.84	3.726667	4.155	6.0625
5810	9.55	7.168571	8.7	4.0675
5811	8.55	4.438	6.461667	1.2125
5812	4.4925	7.31	3.105	4.4275
5813	7.754	6.7	4.816	6.0825

Day 5	Day 6	Day 7
3.7125	3.788	1.374286
5.45	3.622857	2.4025
4.596	3.715	1.94625
4.657143	3.178	2.7075
1.591429	3.014286	2.95125
1.08875	0.71375	0.68125
3.1	2.083333	2.095714
6.598	5.104286	2.97125

Supplemental Figure 2c

Rat ID	Day 1	Day 2	Day 3	Day 4
5804	3	5	4	5
5807	2	2	5	7
5808	3	7	5	5
5809	3	6	4	4
5810	0	7	7	7
5811	0	5	6	8
5812	4	4	6	8
5813	5	4	5	4

Day 5	Day 6	Day 7
4	7	8
7	7	8
5	7	8
7	7	8
7	7	8
8	8	8
6	7	8
7	7	8

Supplemental Figure 3a

Rat ID	Vehicle	DCZ
9901	51.9	57.233
9902	61.9	52.8
9903	50.9	63.9
9905	59.456	56.2
9906	56.7	49.844
9907	46.656	59.9
9908	81.7	67.4
9909	55	57.2

Supplemental Figure 3b

Rat ID	Vehicle	DCZ
9901	7.5	12.8
9902	21.5	12.5
9903	6.3	10.2
9905	5.9	6
9906	5.8	5
9907	4.1	10.3
9908	22.3	28
9909	14.5	16.8

Supplemental Figure 4b

Rat ID	3701	3703	3704	3705	3706
30min	0.4	0.3	0.4	1.8	0.2
60min	0.7	1.3	1	1.8	1.1
2hr	1.1	1.3	1.1	1.8	1.2
4hr	1.7	1.4	1.5	2.6	1.4
6hr	2.2	2.1	2.4	5.4	2.2

Vehicle

3707	3709	3711	3713	3714	3715	3716
0.2	0.2	2.1	2.8	1.3	3.1	2.5
0.2	1.2	2.1	2.9	1.5	3.2	1.6
2.9	1.4	2.2	2.9	1.5	3.2	2.6
3	1.8	4.1	2.9	1.5	3.2	2.6
4.3	3.1	4.5	3.4	2.9	4.4	2.6

					DCZ	
3701	3703	3704	3705	3706	3707	3709
0.5	0.8	2.5	0.1	1.4	3.5	0.7
0.7	3.2	2.7	1.3	1.7	4.4	0.9
1.5	3.7	3.1	3.7	2.1	6.5	2.2
1.7	3.9	3.1	3.9	2.2	7	3.3
1.8	3.9	3.1	3.9	2.5	7	3.4

3711	3713	3714	3715	3716
4.1	4.6	0.5	0.3	3.6
5.2	4.8	0.9	1.2	3.7
5.2	4.8	2.5	1.7	3.9
5.3	4.8	3.6	2.5	4
5.3	4.8	3.6	2.5	4

Supplemental Figure 4d

	Vehicle				
Rat ID	7301	7302	7303	7304	7305
30min	0.3	0.1	0.6	1.3	0.3
60min	0.6	0.2	1.1	1.8	0.3
2hr	1	0.3	1.2	1.9	0.9
4hr	1.3	0.3	2.4	2.3	1.4
6hr	1.4	0.6	2.7	2.3	1.6

						D[illegible]
7306	7307	7308	7301	7302	7303	7304
1	2.1	1.5	0	0.8	2.2	0.6
1.1	2.4	1.7	0.4	0.9	2.4	1.7
2	3.2	3.2	0.4	1.3	3	2.4
3.3	4.6	3.6	0.4	1.8	3.5	3.1
3.6	4.6	3.6	0.4	1.8	3.7	3.1

CZ

7305	7306	7307	7308
0	2.3	1.3	0
0	2.7	2.1	0
0	3.4	2.9	0.8
0	3.7	4	0.8
0	3.9	4.3	1.5

Supplemental Figure 5a

Rat ID	Pre-CS-	Post-CS-
A6501	330.5	440.5
A6502	106.5	854
A6503	1164.5	897
A6504	1278.5	379.5
A6505	740	370.5
A6506	954	970.5
A6507	36.5	554
A6508	660.5	180
A6509	556	695
A6510	648.5	399
A6511	296.5	612.5

Supplemental Figure 5b

Rat ID	Pre-CS+	Post-CS+
A6501	219.5	348
A6502	150	175.5
A6503	603.5	424
A6504	471.5	572
A6505	151	241
A6506	376	275
A6507	151.5	268
A6508	206	209
A6509	167.5	245
A6510	169	234
A6511	581	391.5

Supplemental Figure 5c

Rat ID	ΔCS-	ΔCS+
A6501	110	128.5
A6502	400	25.5
A6503	-267.5	-179.5
A6504	-185.5	100.5
A6505	-369.5	90
A6506	16.5	-101
A6507	350	116.5
A6508	-180	3
A6509	139	77.5
A6510	-249.5	65
A6511	316	-189.5

Supplemental Figure 6a

Rat ID	Day 1	Day 2	Day 3	Day 4
B1801	9.625	0	40.25	87.25
B1802	2.25	25.875	68.25	75.25
B1803	7.125	32.375	86.25	70.25
B1804	4.375	24.75	54.25	15.75
B1805	20.125	35.75	11.375	26.25
B1806	11.75	5.375	20.25	47.75

Day 5
82.875
71.25
52.125
21.125
66.75
70.625

Supplemental Figure 6b

Rat ID	Day 1	Day 2	Day 3	Day 4
B1801	7.725	6.7	4.2875	3.32
B1802	12.03	5.183333	4.908571	2.695
B1803	8.91	10.934	2.917143	3.62
B1804	15.465	8.596667	2.971429	2.89125
B1805	9.29	6.81	6.41	8.558
B1806	11.565	7.91	7.07	6.973333

Day 5
1.32375
2.60875
2.972857
2.9775
4.588
4.87625

Supplemental Figure 6c

Rat ID	Day 1	Day 2	Day 3	Day 4
B1801	2	0	4	8
B1802	1	3	7	8
B1803	1	5	7	8
B1804	2	3	7	8
B1805	2	5	2	5
B1806	2	5	2	6

Day 5

8

8

7

8

5

8

Supplemental Figure 6e

Rat ID	ΔCS-	ΔCS+
B1801	0.1939	0.1261
B1802	0.1723	1.4091
B1803	0.2403	0.8513
B1804	1.0956	0.2567
B1805	0.4958	0.369
B1806	0.5815	0.803

Supplemental Figure 6e

Rat ID	ΔCS-	ΔCS+
B1801	0.0831	0.7117
B1802	0.4073	0.8867
B1803	0.012	0.3613
B1804	0.9152	0.8579
B1805	0.4733	0.9987
B1806	0.7591	1.033
